# Weight-loss intervention using implementation intentions and mental imagery: a randomised control trial study protocol

**DOI:** 10.1186/s12889-015-1578-8

**Published:** 2015-02-27

**Authors:** Anne Hattar, Martin S Hagger, Sebely Pal

**Affiliations:** Health Psychology and Behavioural Medicine Research Group, School of Psychology and Speech Pathology, Faculty of Health Sciences, Curtin University, GPO Box U1987, Perth, 6845 Western Australia Australia

**Keywords:** Behaviour change, Calorie counting, Cost effectiveness, Dietary intake, Exercise, Goal reminder text messages, Health action process approach, Implementation intentions, Mental imagery, Physical activity, Obesity, Overweight, Short message service, Weight-loss

## Abstract

**Background:**

Overweight and obesity are major health problems worldwide. This protocol describes the HEALTHI (Healthy Eating and Active LifesTyle Health Intervention) Program, a 12-week randomised-controlled weight-loss intervention that adopts two theory-based intervention techniques, *mental imagery* and *implementation intentions*, a behaviour-change technique based on planning that have been shown to be effective in promoting health-behaviour change in previous research. The effectiveness of goal-reminder text messages to augment intervention effects will also be tested. The trial will determine the effects of a brief, low cost, theory-based weight-loss intervention to improve dietary intake and physical activity behaviour and facilitate weight-loss in overweight and obese individuals.

**Methods/Design:**

Overweight or obese participants will be randomly allocated to one of three conditions: (1) a psycho-education plus an implementation intentions and mental imagery condition; (2) a psycho-education plus an implementation intentions and mental imagery condition with text messages; or (3) a psycho-education control condition. The intervention will be delivered via video presentation to increase the intervention’s applicability in multiple contexts and keep costs low. We hypothesise that the intervention conditions will lead to statistically-significant changes in the primary and secondary outcome variables measured at 6 and 12 weeks post-intervention relative to the psycho-education control condition after controlling for baseline values. The primary outcome variable will be body weight and secondary outcome variables will be biomedical (body mass, body fat percentage, muscle mass, waist-hip circumference ratio, systolic and diastolic blood pressure, low-density lipoprotein, high-density lipoprotein, total cholesterol, triglycerides, blood glucose and insulin levels), psychological (quality of life, motivation, risk perception, outcome expectancy, intention, action self-efficacy, maintenance self-efficacy, goal setting and planning), and behavioural (self-reported diet intake, and physical activity involvement) measures. We also expect the intervention condition augmented with text messages to lead to statistically significant differences in the primary and secondary outcome variables at the follow up periods after controlling for baseline values.

**Discussion:**

The planned trial will test the effectiveness of the theory-based HEALTHI program intervention to reduce weight and salient psychological, biomedical, and behavioural outcomes in overweight and obese adults. The study has been designed to maximise applicability to real world settings and could be integrated into existing weight management practices.

**Trial registration:**

ACTRN: ACTRN12613001274763. Registration date 19/11/2013.

## Background

### The problem of overweight and obesity

Obesity and overweight are linked to major chronic illnesses and conditions like cardiovascular disease, diabetes, and certain cancers [1]. It is recognised as a global problem with obesity rates worldwide having nearly doubled since 1980 [1]. This has led researchers to develop effective means to assist obese and overweight individuals to reduce their weight and, in doing so, their risk of chronic illness. The main cause of weight gain is an imbalance between the amount of energy consumed and expended by individuals. Wing and Hill [2] report that weight-loss can only be achieved through a negative energy balance, decreasing energy intake by restricting food or increasing energy expenditure through daily activity. Based on current evidence, weight-loss strategies that include a combination of a reduced calorie intake diet and increased physical activity appear to be most effective [3,4]. The current article will discuss the development of a brief, cost effective, theory-based intervention using implementation intentions and mental imagery techniques to target dietary intake and physical activity behaviour to promote weight-loss. The intervention will involve minimal direct contact between participants and practitioner and will use resources that can easily be ‘rolled-out’ in ‘real world’ contexts. The intervention will use the Health Action Process Approach (HAPA) [5] as a theoretical basis for intervention content and to explain the mechanisms behind the proposed effects. The intervention will also incorporate the use of goal reminder text messaging to augment the intervention effectiveness.

### Diet, physical activity, and weight loss

Research suggests that combining a calorie-reduced diet and regular physical activity will be effective in achieving weight loss and preventing obesity related risk factors [3,6-9]. Reducing 500 calories per day can assist with a weight-loss of approximately 0.5 kilograms a week [10,11]. Fernau [10], suggests that one strategy that individuals may use to manage the consumption of food is to count daily calories. Some of the methods to assist with calorie counting are to spread calories throughout the day, read labels including the serving size and calories per serving, use a calorie counter such as a book or website, and monitor daily food and drink intake such as through the use of a food diary (*see* Fernau 2010, for more information) [10].

Engaging in regular physical activity is also an important part of an effective strategy to assist an individual to expend more calories than the body requires thereby contributing to weight loss. Exercise can assist in improving risk factors associated with cardiovascular disease even in the absence of weight-loss [7,9,12,13]. Physical activity guidelines in America and Australia recommend that adults engage in a minimum of 30 minutes of moderate activity on most or all days of the week to receive health benefits [14]. A combination of changes in dietary intake and physical activity behaviour will likely be most effective in assisting an individual to lose weight [3,4].

### Changing health behaviour

For health professionals to support clients to lose weight, it is necessary to identify the factors related to health-related behaviour change and use them as a basis for intervention design [15,16]. These factors are outlined in many different behaviour change models, particularly social psychological models which aim to explain intentional and motivated behaviour that have been applied to predict and understand health behaviour [17]. Researchers seeking behavioural solutions to health-behaviour need to target psychological factors that are considered changeable and malleable to evoke health-behaviour change. These psychological factors can be targeted by sets of behaviour-change techniques that can evoke a change in the psychological factors linked to health behaviours [18,19].

**Figure 1:**
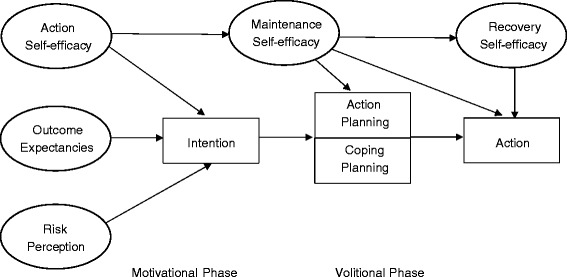
**Health action process approach model (Schwarzer, 2008).**

The current intervention adopts the HAPA [5], an integrated social psychological model that has been widely used to identify the components related to changes in weight loss behaviours and how components can be used to inform the intervention content to ensure the change process is clearly identified (see Figure 1). The HAPA states that the process of health behaviour change consists of two phases: a motivational phase that leads an individual to form the intention to change their behaviour, and a volitional phase that leads the individual to performing the actual behaviour [5]. Two strategies that map closely on to the volitional and motivational phases of the HAPA include implementation intentions and mental imagery, respectively. The intervention components are designed to target both the motivational and implemental phases of the HAPA. The components are proposed to lead to optimal behaviour change by providing individuals with the confidence and motivation to engage in the behaviour, as well as the capacity to convert motivation into actual behaviour change [20].

Mental imagery has been found to increase an individual’s confidence in their ability to change their behaviour (self-efficacy), which relates to the motivational phase in the HAPA. Mental imagery can be categorised as either being outcome imagery or process imagery, both which have different implications for goal attainment [21-23]. Outcome imagery is based on imagery related to the experience of goal achievement and success, and therefore is a motivational approach for improving goal achievement. Process imagery is based on imagery related to the steps needed to achieve the desired outcome, which according to Pham and Taylor [24] is mainly a cognitive planning strategy [23]. Implementation intentions are action plans that state when, where, and how an individual intends to reach a goal and promotes action planning, bridging the gap between having the intention to change behaviour and performing the behaviour, consistent with the volitional phase of the HAPA [20,25]. To target changes in diet and physical activity behaviours for weight-loss, the combined use of implementation intentions and mental imagery have been found to be an effective method [26].

Mobile phone text messaging is a widely available, cost effective, and an instant tool that has been recently applied to promote health behaviour change and to increase intervention effectiveness [27]. Support for the use of text messages to increase intervention effectiveness is found within various studies exploring the outcome of weight-loss [28-31]. For example, Prestwich and colleagues [31] developed an intervention aimed in promoting brisk walking among sedentary individuals. The researchers found that pairing implementation intentions with goal reminder text messages assisted participants in increasing walking frequency and weight loss. Text messages which include goal reminders may enhance the effectiveness of both implementation intentions and mental imagery steps, respectively within the volitional and motivational phase of the HAPA; The use of text message goal reminders can serve to “boost” the effectiveness of these intervention components as that may assist participants in recalling their goals and plans increasing their commitment to action them, as well as increasing ones confidence to change their behaviour.

## The present study

The purpose of the current study is to develop and evaluate the HEALTHI (Healthy Eating and Active LifesTyle Health Intervention) program, a theory-based intervention to change dietary intake and physical activity behaviours, and promote weight-loss and the reduction of obesity-related health risk factors in a sample of overweight and obese individuals. The key behavioural outcome will be changes in body weight through engagement in, and adherence to, a calorie-restricted diet and participation in 30 minutes of daily physical activity. Although a number of studies have incorporated the use of implementation intentions and mental imagery to promote health behaviour [21-23,32] the present intervention will incorporate the use of both strategies to promote both diet and physical activity guidelines adherence. The inclusion of both strategies acting synergistically with multiple longer-term follow-up of biomedical, behavioural, and psychological outcomes is a unique aspect of the current intervention and will contribute to knowledge by demonstrating whether these techniques are effective in bringing about weight loss and other outcomes changes.

The present intervention will also incorporate the use of technology, augmenting the effectiveness of the intervention with goal-reminder text messages delivered by mobile phone. The use of multiple strategies in achieving weight loss goals including implementation intentions, mental imagery and text message goal reminders is a unique aspect of the current study. Pairing implementation intentions with text message goal reminders has been shown to be successful for weight-loss and participation in physical activity [31]; the aim of the study is to not only deliver implementation intention, but also mental imagery goal reminder text messages based on the HAPA. This is important as research has shown that only a few studies using text messaging for behaviour change in disease prevention and treatment had a theoretical rationale to guide various interventions [27]. This will be the first study to demonstrate the use of both text messaging with implementation intention and mental imagery in weight management. This will address a specific gap in the research literature regarding weight management and will contribute to knowledge by testing the proposal that introducing multiple theory-based behaviour-change strategies alongside a standard weight-change intervention program will improve the effectiveness of the intervention [33,34].

### Hypotheses

The main hypothesis is that the intervention conditions adopting implementation intentions and mental imagery techniques, will result in statistically-significant independent improvements in the outcome measures relative to the control condition from the time of intervention and at 6 and 12 weeks. We also expect statistically-significant differences in the outcome variables across the two intervention conditions, such that the intervention condition with text messages leads to greater weight loss that the intervention condition alone.

It is also hypothesised that relative to baseline and week 6 and 12, the intervention with text message condition will lead to statistically-significant independent improvements on the biomedical, psychological, and behavioural outcome measures, compared with the intervention and control conditions. In particular, we expect to find statistically-significant differences in the key HAPA-based psychological mediators of the effects of the intervention components including planning (relevant to implementation intentions) and self-efficacy and motivation (relevant to mental imagery) over the same follow-up time points.

## Methods and design

### Design

**Figure 2:**
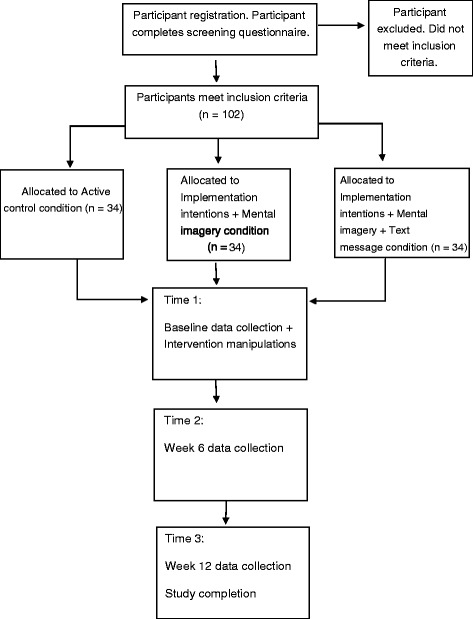
**Participant flow diagram.**

A randomised controlled design will include obese and overweight participants randomly allocated to one of three conditions: A psycho-education control condition (control condition), an implementation intention and mental imagery techniques condition (intervention condition), and an implementation intention and mental imagery techniques with text messages condition (intervention with text message condition). Figure 2 displays the design of the intervention and proposed participant flow through the study. The 12-week study will examine the effects of the intervention on biomedical, psychological, and behavioural outcome measures at baseline and post intervention (at 6 and 12 week follow-ups). The study information and intervention manipulations will be delivered via a video presentation viewed at baseline. See Table 1. While viewing the video intervention manipulations participants will be asked to complete various activities such a goal setting. The responses to the intervention activities will be emailed to the research team to indicate program compliance and adherence. Ethical approval for the trial has been obtained from Curtin University’s Human Research Ethics (approval number: HR137/2013). The trial has been registered on ANZCTR (Trial number: ACTRN12613001274763).

**Table 1:** **Study components specific to each condition**

**Conditions**		
***Control***	***Intervention***	***Intervention with text message***
● Study information	● Study information	● Study information
● Psycho-education	● Psycho-education	● Psycho-education
● Weight loss guidelines	● Weight loss guidelines	● Weight loss guidelines
● Goal setting	● Goal setting	● Goal setting
	● Implementation intentions	● Implementation intentions
	● Mental imagery	● Mental imagery
		● Text messages

### Participants

Participants will be recruited via the Curtin University website and email system, flyers in local community organisations, a Western Australian Newspaper and a social-networking website. Eligible respondents recruited via these procedures will be provided with study information and an online link to a corresponding online questionnaire (Qualtrics™). Participants will be directed to complete the online screening questionnaire to ensure they meet the study inclusion criteria. Participants are eligible for inclusion if they are aged between 18 and 65 years, overweight or obese, defined as a body mass index (BMI) of between 25 and 40, and cite a willingness to have blood samples and body composition scans taken. Study exclusion criteria include serious health conditions, inability to engage in any form of physical activity, pregnancy, severe depression, inability to commit to follow up clinic sessions, or no access to a mobile phone with text message features. A comprehensive list of the inclusion and exclusion criteria is provided in Appendix A.

Participants that meet the study inclusion criteria will be invited to attend an initial orientation session at the Curtin University clinic to provide them with study information. Individuals are then given an opportunity to officially enrol in the program at the orientation session. Prior to their first clinic appointment the participants will be directed to complete baseline measurements.

## Intervention protocol

### Procedure

The study will adopt a double-blind procedure so that both the participant and researcher are unaware of the intervention conditions to which they have been assigned. A research assistant naïve to the purpose of the study will ensure each participant is provided with a participant identification number and randomly allocated to one of the three conditions using a computer-generated schedule [35].The intervention information and procedures for each of the three conditions will be determined by the allocated condition and participants will be required to access the video presentation corresponding to their allocated condition. Participants in the intervention with text message condition, will have their participant identification number matched to their mobile phone number. The mobile numbers will be entered on a text message system to allow the researcher to send participants their weekly text message goal reminders.

Participants will need to provide written consent prior to participation. Prior to each clinic appointment participants will complete questionnaires measuring baseline psychological and behavioural outcomes including a 3 day food, drink, and exercise diary. Within a three day period, prior to their clinic appointment, participants will be required to attend to a pathology clinic to take blood sample measures. Furthermore, at each clinic appointment participants will engage in baseline biomedical assessments: blood pressure, weight, waist-hip ratio, and at baseline and week 12 body composition measures. At the first clinic appointment participants will be asked to view a pre-recorded video presentation that contains the intervention information, followed by the completion of a questionnaire containing the post-intervention measures. One of two video presentations will be provided to participants, one designed for the control condition participants, and the other for the two intervention conditions. The duration of the video presentation is approximately 60 and 76 minutes for the control and intervention conditions respectively. Due to the pauses in the video to complete various activities and the break time the intervention manipulation duration may last approximately 90 to 120 for the control condition, and 150 to 210 for the intervention conditions. In addition, participants allocated to the intervention with text message condition will receive goal reminder text messages over the 12 weeks of the intervention. Prior to leaving the clinic, all participants will be provided with their future appointment times and a set of tools to assist them with monitoring their physical activity and calorie intake: A copy of the Calorie King™ book [36], Calorie Counter diary [37], the Australian dietary guidelines [38], and Australian physical activity guidelines [39]. Participants will be encouraged to contact the researcher if any queries arise. In addition, a personalised email providing a handout of the essential program information such as the guidelines and the participant’s responses to various activities (including goals) when watching the video presentation will be sent to the participant via email. The participant responses to the activities will be assessed by the research team to ensure program compliance.

## Intervention

### Weight-loss information for all participants

Participants will be presented with a psycho-educational component common to all intervention conditions followed by, if assigned to the intervention conditions, the behaviour change components based on the HAPA. The components will be delivered by an interactive video presentation by an investigator. Table 1 outlines the components specific to each of the three study conditions.

The significance of adopting theory-based intervention techniques delivered by video means that the intervention is evidence-based, cost effective, requires minimal contact with the participant, provides flexibility for delivery, and is easily available. The video presentation allows messages to be conveyed in a consistent manner and reduces experimenter bias and provides greater control over potential variation in delivery across participants. All participants will receive identical content in relation to study design, psycho-education information, dietary intake and physical activity guidelines. Thus differences between the control and intervention conditions can be attributed purely to the intervention components. The participants in the two intervention conditions will receive the same information regarding the mental imagery and implementation intentions instructions, and thus differences in these intervention conditions can be attributed to the goal reminder text messages and not differences in intervention delivery style.

The main components of the video presentation are outlined below^a^. The pre-recorded video presentation provided to participants within all conditions, will introduce participants to study information and basic concepts of weight loss including psycho-education. The participants will be informed that they will be required to adhere to two guidelines for physical activity and dietary intake, respectively, in accordance with the weight-loss intervention.

The physical activity guideline encourages participants to exercise and be physically active. Participants will be instructed to “*engage in physical activity and be as active as possible. Engage in a minimum of 30 minutes of planned daily exercise*” in accordance with the Australian physical activity guidelines [39]. Participants will be provided with examples of physical activity and exercise, and be informed that more intensive exercise will assist them in reaching their *weight-loss* goal. Participants will be instructed to record some goals related to the physical activity guidelines.

The dietary intake guideline encourages participants to monitor their energy intake. The participants are instructed: “*monitor your energy intake and ensure your calorie (or kilojoule) intake amount is somewhere in-between the minimum and maximum allowance”.* Each participant will be provided with an individually recommended daily calorie intake amount needed for weight-loss. The Katch-McArdle Formula will be used to calculate the minimum amount of energy needed for one’s body to function, or the calories needed to maintain ones weight, referred to as basal metabolic rate (BMR), [40]. The Katch-McArdle formula takes into consideration an individual’s lean body mass. (BMR = 370 + (21.6 × lean mass in kg). Total daily energy expenditure is calculated by multiplying the BMR to a number representing the individual’s physical activity level. The outcome is subtracted by 500 calories to provide individuals with the recommended number of calories needed to consume to achieve weight-loss [10]. Participants’ calorie intake allowance will be recalculated at week 6 to account for body weight changes. To assist in meeting their daily recommended calories allowance, participants will be provided with information on the definition of a calorie, information on calories versus kilojoules, how to calorie count, how to use a calorie counting book, suggestions on food and drink consumption to assist in remaining within a participant’s daily calorie allowance, how to ensure a healthy diet including information on the Australian dietary guidelines [41], and examples of healthy eating. Participants will be instructed to record some goals related to the dietary intake guidelines.

### Components for intervention conditions

The pre-recorded video presentation provided to participants in the intervention conditions will also include the mental imagery and implementation intentions intervention components. Participants will be asked to create implementation intention plans using an if-then format [20]. Participants will be provided with instructions to guide them to mentally imagine the steps needed to reach their goals, and the goal outcome. Participants will be directed to record their goals, if-then plans, and mental imagery steps. Information on the development of the behaviour-change intervention components based on the HAPA is outlined below.

Research has highlighted the importance of outlining details of the behaviour change techniques used in behavioural interventions in relation to how they fit the theory [18,33,42,43]. This allows for an understanding of the content that an intervention uses, the methods, and the ‘active ingredients’ that lead to behaviour change. Michie and Prestwich [44] also highlight the importance of not only using theory to guide an intervention, but also identifying the theory components that relate to the intervention techniques.

Table 2 provides details of our systematic method of matching the study techniques with the theoretical components; this allows for better understanding of how the theory informs the intervention design and assists in evaluating the intervention effectiveness and theory [42,44]. The table includes the behavioural objective or target behaviour to be changed such as the diet or physical activity guideline, the theory-based psychological concept employed, the behaviour change technique within the theoretical framework that relates to the objective, the strategies that will be used in the present study linking the performance objective and behaviour change technique, and the evaluation method, such as the questionnaires or tools that will be used to evaluate whether the strategies employed resulted in changes in the theoretical construct to which it pertained.

**Table 2:** **Systematic process matching behaviour-change technique with theoretical components for the HEALTHI program**

**Objective/Behaviour**	**Psychological variable targeted**	**Behaviour change technique (theoretical framework)**	**Implementation strategies**	**Evaluation**
1. Diet: *Monitor your energy intake and ensure your calorie (or kilojoule) intake amount is somewhere in-between the minimum and maximum allowance amount.*	Risk perception	Information provision.	Provide information on obesity related health factors.	Questionnaire items measuring Risk perception.
	Planning	Implementation Intentions	Participants create If-then plans on: how they plan to count calories, and how to keep calories to a minimum.	Questionnaire items measuring planning and level of If-then plan.
	Self-efficacy and motivation	Mental Imagery	Participants are directed to imagine the steps needed to reach their If-then plan.	Questionnaire items to measure self-efficacy and motivation.
			For example, imagining that they want to have a fatty food, and then imagine the steps required to implement their implementation intentions that target this obstacle.	
2. Physical Activity: *Engage in physical activity and be as active as possible. Engage in a minimum of 30 minutes of planned daily exercise.*	Risk perception	Information provision	Provide information on obesity related health issues and benefit of physical activity.	Questionnaire items to measure risk perception.
	Planning	Implementation Intentions	Participants create If-then plans on how they will engage in exercise, and what exercise they will perform.	Questionnaire items to measure planning and level of the If-then plan.
	Self-efficacy and motivation	Mental Imagery	Participants are directed to imagine the steps needed to reach their If-then plan.	Questionnaire items to measure self-efficacy and motivation.
*Information on objectives needed for both Goal 1 (dietary intake) and Goal 2 (physical activity)*	Evaluation	Self-evaluation	Self-monitor dietary intake and physical activity involvement.	Food, drink, and exercise diary.
				Questionnaires to measure dietary and physical activity behaviour.
				Body weight measurements.
	Outcome expectancies (subjective beliefs about unforseen events of a person’s behaviour with succeeding outcomes. That is, a person’s expectations about the consequences of an action).	Information provision.	When changing behaviour, the outcomes are viewed as being negative or positive (e.g., weight-loss) and this influences intention to engage in target behaviour (exercise, diet).	Questionnaire items to measure outcome expectancy.
			Information on positive outcomes of weight-loss and engaging in diet and exercise.	

Delivery mode = intervention delivered via online Qualtrics™ with incorporated video presentation.

One condition will receive goal reminder text messages.

Intensity time = participants are asked to view the intervention once and complete the various activities incorporated within the intervention.

Setting = Clinic room within Curtin University.

For example, the objective *“*monitor your energy intake to ensure your calorie (or kilojoule) intake is somewhere in-between the minimum and maximum allowance amount” is listed as the behavioural objective or behaviour needed to be changed (Table 2). The intervention components are based on theory-based psychological concepts: risk perception and planning. The planning variable target is matched with the behaviour change techniques of implementation intentions. The implementation intentions relate to the use of the strategy of directing participants to create if-then plans in relation to the diet guideline. The strategy will be evaluated by having participant’s complete self-reported questionnaires on planning and the if-then plan will be evaluated by the researcher.

#### Text message intervention component

Participants in the intervention with text message condition, will receive a text message once a week for a total of twelve weeks. The text message content will include goal reminders that relate to the participant’s diet and physical activity goals, implementation intentions and mental imagery that were set at baseline. For example: “Hello. I would like to ask you to take the time to review and remember your goals and the steps you need to take to reach them. Try and remember (or refer to your HEALTHI Program handouts) when you imagined the steps for your dietary intake and physical activity/exercise goals when watching the video? Take a few minutes to imagine these steps to reach your goals so it is clear in your mind”.

## Assessments

Multiple measures of biomedical, psychological, and behavioural outcomes and measures will be used to assess the intervention effectiveness; these are discussed below.

### Biomedical outcomes

Key outcome biomedical outcome measures will be taken to monitor changes in cardiovascular disease risk and body weight and composition as a result of the intervention [7,9,45]. All the biomedical outcome measures will be conducted at baseline, 6 and 12 week periods unless specified otherwise.

Within a three day period prior to the clinic appointment, blood lipoproteins including low-density lipoprotein (LDL), high-density lipoprotein (HDL), total cholesterol (TC), triglycerides (TG), and blood glucose and insulin levels will be taken at an approved pathology laboratory using a venous blood sample from each participants.

*Body weight (kg)* will be recorded with participants wearing light clothing and no shoes, using a digital body composition monitor scale (Omron, model HBF 362). *Height* (cm) will be measured at baseline, to the nearest 0.1 cm using a stadiometer with participants wearing light clothing and no shoes. *Body mass index* (BMI) will be calculated using both the weight and height measurements using the formula weight in kilograms divided by the square of the height in meters (kg/m^2^). *Waist-hip ratio* (cm) will be calculated by measuring waist circumference in standing position at the narrowest area between the iliac crest and lateral lower rib, and hip measurement from the largest circumference of the lower abdomen. This will be measured to the nearest 0.1 cm using a circumference measuring tape (Seca 203). To ensure greater accuracy the measure will be taken twice and the average of the two readings will be used.

*Systolic blood pressure* (SBP) and *diastolic blood pressure* (DBP) will be measured using an automated, blood pressure monitor (A & D Medical, model UA-851) with participants in a supine position with the tested arm at the level of their heart for at least a minimum of 10 minutes before and during measurements [46]. The measure will be taken three times and the average reading will be used. Body composition including *bone mass, body fat percentage,* and *muscle mass* will be measured using the whole body dual-energy X-ray absorptiometry (DEXA, Lunar Prodigy; Lunar, Madison, WI, USA). Participants will be required to lie on a bed during the DEXA scanning with no jewellery or metal clothing. DEXA scans will be performed at baseline and 12 weeks. The dual-energy X-ray absorptiometry machine will be calibrated and a phantom scan undertaken daily.

### Behavioural Outcomes

A number of behavioural outcome measures will be taken at baseline, 6 and 12 weeks, unless otherwise specified. The 24-item Bailey Dietary Screening Questionnaire will be used to identify nutritional risk. Three levels of nutritional risk include: at risk, possible risk, and not at risk. The measure has been found to have adequate sensitivity and specificity when compared with nutritional risk based on dietary reference intakes [47]. Sensitively measures indicate that 83% of individuals were correctly classified as positive by the screening tool. Specificity measures indicated 75% of individuals correctly classified as negative by the screening tool. An example item from the questionnaire is: “how often do you usually eat fruit as a snack?” Participants are asked to respond on a Likert scale ranging from 0 (never) to 4 (3 or more times a week).

An 18 item version of the *Three Factor Eating Questionnaire*, (TFEQ-R18), will be used to measure eating behaviour examining three cognitive and behavioural domains of eating; these include cognitive restraint, uncontrolled eating, and emotional eating. Good reliability has been reported [48]. An example of an item within the emotional eating scale includes “I start to eat when I feel anxious”. The item response occurs on a 4 point Likert scale from 1 (definitely true) to 4 (definitely false).

### Physical activity and diet intake diary

Prior to each clinic visit, participants will complete a 3 consecutive day record of their physical activity and dietary intake. Participants will complete the ‘food, drink and exercise diary’ record including 1 weekend and 2 week days [49]. For the physical activity section, participants will complete a 3-day activity log adapted from Bouchard and colleagues [50]. Participants will record an activity code from 1 to 9 that corresponds to the physical activity carried out during each five minute period; the time and activity of physical activity is thus recorded. In addition, participants will record their belief about the accuracy of their physical activity log. The physical activity log information will be assessed by examining energy expenditure. Each activity code is related to various levels of Metabolic Equivalent. The measure has been found to have high reliability with a coefficient of 0.96. For the food diary intake section, participants will record a 3-day food log containing information about their drink or food intake, time consumed, location, amount, drink or food description and method of preparation, and belief about the accuracy of their food log. The diary will be used to evaluate the participant’s compliance with the recommended diet and physical activity guidelines. The program Food Works will be used to analyse the food content and nutrients [49].

*Self-reported intervention compliance questions* were also incorporated at week 6 and 12. The 5 items will be used as a measure of the participant’s self-reported compliance to the behaviour changes of dietary intake and physical activity. For example, “on average, during the past 6 weeks, how often did you engage in at least 30 minutes of planned exercise?” with item responses ranging from 1 (7 days/week or Always) to 5 (Never).

### Psychological measures

Participants will be instructed to complete the outlined psychological measures before and after viewing the video presentation intervention unless specified otherwise. Variables from the HAPA will be measured to assess the psychological mediators that may explain the effects of the intervention on the behavioural and biomedical outcomes. The HAPA measures include items adapted from Barg and colleagues’ [51] measure of assessing risk perception for developing cancer for physically inactive middle aged adults. *Risk perception* will be assessed using four items on a 6 point Likert scale ranging from 1 (strongly disagree, much lower, not likely) to 6 (don’t know/refused) to measure perceived risk of developing obesity related risk factors. For example, “I think it is likely that I will develop health problems related to obesity at some point in my life.” *Outcome expectancy* will be assessed using six items with responses ranging from 1 (strongly disagree, not at all effective) to 5 (strongly agree, extremely effective) to question participants about the effect of diet and physical activity on health risks related to weight gain. For example, “I think that consuming fewer calories per day is a very important way to help me to lose weight”. *Intention* will be assessed with four items that ask participants how much they plan and intend to follow the diet guidelines and/or to participate in the recommended physical activity amount over the next 12 weeks. Responses range from 1 (strongly disagree) to 5 (strongly agree). For example, “I intend to participate in daily physical activity with a minimum of 30 minutes of planned exercise on each individual occasion over the next 6 weeks. *Action self-efficacy* will be measured using 10 items regarding participants’ confidence and ability to engage in the recommended diet and physical activity guidelines over the next 12 weeks. Prior to answering the questions, participants would have heard the recommendations during the video presentation. Items are on a 5 point Likert scale ranging from 1 (not confident, not likely, strongly disagree) to 5 (completely confident, extremely confident, strongly agree). For example, “if it were entirely up to you, how confident are you that you would be able to follow a diet that requires you to consume fewer calories per day on each individual occasion over the next 6 weeks?” *Maintenance self-efficacy* will be measured using 18 items on a 5 point Likert scale ranging from 1 (not confident) to 5 (completely confident) to measure barriers to engage in the recommended physical activity or diet guidelines. For example, participants were asked “How confident are you that you will do daily physical activity with a minimum of 30 minutes of planned exercise during your leisure time on each individual occasion over the next 6 weeks even if…” followed by a list of barriers, such as, but not limited to bad weather and feeling tired. The participants *planning* will be assessed using two items ranging from 1 (strongly disagree) to 5 (strongly agree) that ask participants if they had made a detailed plan about when, where and how they would engage in physical activity or follow the diet guidelines. For example, “I have made a detailed plan about when, where, and how I will do daily physical activity with a minimum of 30 minutes of planned exercise on each individual occasion over the next 6 weeks”.

Further variables that relate to the HAPA were adopted from Hagger and colleagues [32]. *Motivation* will be assessed at baseline by asking participants to respond to six items that question motivation to participate in physical activity and change their diet. For example, “how motivated are you to change your diet on each individual occasion over the next 12 weeks?” Item responses are based on a 6 point Likert scale ranging from 1 (not at all motivated, no effort at all) to 6 (extremely motivated, all my effort). In addition four items exploring engagement in mental imagery exercises will be used. Participants will be asked to indicate the level to which they were able to imagine the task. For example, “to what extent did you visualise in your mind (imagine) exactly how you might reach your diet goals (example, consuming fewer calories per day) on each individual occasion over the next 6 weeks?” The items are on a 6 point Likert scale ranging from 1 (indicating did not imagine), to 6 (indicating clearly imagined).

*Goal setting* will be measured by asking participants six open ended questions. For example, ‘What are your goals for physical activity?’ The answers to these questions will be “coded” appropriately based on whether they are appropriate and realistic. *Intervention checks* will also be included to ensure that participants engaged with and completed the implementation intention and mental imagery exercises appropriately and according to protocol. That is, participants will be asked to record their goals; If-then plans; and a list of the steps they imagined during the intervention process. The answers to these questions will be “coded” appropriately based on whether they are appropriate and realistic. These variables will be measured as they are expected to mediate the key components of the intervention. That is, the effect of the mental imagery component of the intervention variable on the primary and secondary outcomes is expected to mediate the HAPA variables of self-efficacy, motivation, outcome evaluation, and intention. Similarly, the effect of the if-then plan component of the intervention on the primary and secondary outcomes is expected to be mediated by the HAPA planning variable.

Imagery ability will be measured at baseline as it may moderate the effectiveness of the mental imagery intervention and should therefore be included as a potential covariate. Imagery ability will be measured using the *Betts Questionnaire upon Mental Imagery* [52] is a 35 item questionnaire used to measure the clarity and vividness of their mental imagery ability. The questionnaire instructs participants to imagine, for example “Visualize a mental image of a friend you see on a regular basis” and rate the image vividness on a Likert scale. Sheehan suggests that the measure is valid and has predictive value. The scale has also been found to be reliable [53].

The variables of quality of life, depression, anxiety, and stress will be measured as a control variables to ensure the intervention does not have adverse effects on quality of life or emotion. These variables will be measured using the two listed questionnaires. *The Impact of Weight on Quality of Life Questionnaire (IWQOL - Lite)* is a 31 item version, self-reported measure that assesses the effect of obesity on quality of life in five domains: physical function, self-esteem, sexual life, public distress, and work [54]. For example, a physical function item includes “because of my weight I have difficulty picking up objects” with responses made on 5 point Likert scales ranging from 1 (never true) to 5 (always true). *Depression Anxiety Stress Scales-21* (DASS-21) will be used to measure symptoms of depression, anxiety, and stress. At baseline, 6 and 12 weeks, participants will rate their symptoms over the past week by answering 21 items using a 4-point Likert Scale anchored by 0 (did not apply to me at all) and 3 (applied to me very much, or most of the time) [55]. An example item from the questionnaire includes “I couldn’t seem to experience any positive feeling at all”.

#### Statistical analysis

A series of 3 (condition: control, intervention, and intervention with text message) × 2 (time: 6 and 12 weeks) mixed model multivariate analyses of covariance (MANCOVAs) with time as a repeated measures variable will be used examine the effects of the intention over time on study outcome variables. Baseline measures will be covariates in the model. A separate MANCOVA will be conducted for each set of dependent outcome variables: biomedical, behavioural and psychological. Pending significant multivariate effects, follow-up univariate analysis of covariance (ANCOVAs) will be used to identify differences for individual variables within each outcome set. Post-hoc follow-up tests will also be conducted to locate individual differences at the time intervals. Mediation analyses will be conducted by mediated multiple linear regression analysis with dummy-coded intervention conditions as the independent predictor variables, the constructs from the HAPA included as mediating variables, and the primary and secondary biomedical outcome variables as the dependent variables. Mediation will be confirmed through significant bootstrapped indirect effects using Preacher and Hayes’ [56] algorithms for multiple mediation and the PROCESS macro. Missing data will be handled by multiple imputation using regression analysis with the Markov-chain Monte Carlo (MCMC) estimation method. In addition, we will adopt a full intention-to-treat analysis with last measured data points carried forward in order to provide a conservative estimate of the effects.

Sample size has been determined by a power analysis using the G*Power 3.1 Program, suitable to evaluate differences between the three condition levels greater than 10%, with statistical power set at > 0.80 and alpha set at *p* < .05 [57]. A large effect size is assumed as previous studies that have used implementation intentions and reminder text messages [31], as well as studies using mental imagery and implementation intentions to increase exercise behaviour [58], have found large effect sizes. Given the estimated effect size, the total sample size required post-test is 66 and considering a potential 35% drop out rate and eliminations of cases due to missing data or spoilt questionnaires based on previous trials [21], we plan to recruit 102 participants at baseline or until the required sample size has been achieved.

## Discussion

The purpose of the current intervention is to examine the effectiveness of an intervention using implementation intentions and mental imagery strategies aimed to promote adherence to diet and physical activity guidelines to assist obese or overweight individuals in achieving weight loss. The intervention will also evaluate whether mobile phone goal reminder text messages will be effective in augmenting the intervention effectiveness. The randomised control trial will consist of participants being randomly allocated to one of three conditions: (1) a psycho-education plus an implementation intentions and mental imagery condition; (2) a psycho-education plus an implementation intentions and mental imagery condition with text messages; or (3) a psycho-education control condition. The intervention will be delivered via video presentation, an approach that will increase the intervention’s applicability in multiple contexts and keep costs low. The research will be guided by the theoretical framework offered by the HAPA to explain the effects of the intervention components and the mechanisms and mediators involved. The key behavioural outcomes will be adherence to a calorie restricted diet and participation in 30 minutes of daily physical activity measured at 6 and 12 weeks post-intervention. The intervention will also explore changes in additional key biomedical (for e.g., body fat, and waist-hip circumference), psychological (for e.g., quality of life, motivation, and planning), and behavioural (for e.g., self-reported diet intake and physical activity involvement) health-related outcomes.

A large number of questionnaires and assessments completed by participants may have the potential to introduce considerable response burden on participants and lead to increased measurement error and affirmation bias. Furthermore, it is possible that only motivated and potentially compliant people will be likely to persist with the study introducing bias to the eventual findings. As with all research the number of measurements selected is a trade-off between collecting adequate data to evaluate the effectiveness of the intervention relative to participant burden and demand. However, it must be stressed that we have put a number of strategies in place to manage the issue of participant burden and allay associated confounding effects. Specifically, during the video intervention manipulation participants will be encouraged to take as many breaks as needed and have the opportunity to pause the video at their own volition. In addition, following the first appointment most of the measures and assessments will only be completed relatively infrequently i.e. at six-week intervals.

A unique aspect of the present study is the adoption of two key theory-based intervention techniques, implementation intentions and mental imagery, in synergy to assist participants in achieving their weight loss goals. The inclusion of these components together is consistent with contemporary theory based on the HAPA that two phases to action exist and the current intervention aims to induce behavioural engagement through the inclusion of components targeting both phases. The theory-related components and concepts outlined in the current protocol are explicitly linked using a systematic intervention-matching procedure providing other researchers with a precise account of the intervention content and its theoretical basis. The current research is also unique as it will examine whether augmenting the intervention using text messages by mobile phone will enhance the effectiveness of the intervention messages. Prestwich and colleagues [31] paired implementation intentions with text message goal reminders to achieve participant weight-loss and participation in physical activity the current study extends this research by including the motivational intervention component of mental imagery alongside implementation intentions with text messages.

Another unique component of the current intervention is the adoption of a streamlined and efficient study design with low administration and personnel required with intervention delivery being via video presentation. The significance of implementing a theory based intervention via video presentation means that the intervention is evidence based, cost effective, requires minimal contact with the participant, provides flexibility for delivery, and is easily available. The intervention may provide health professionals with a means of assisting obese or overweight individuals that seek weight-loss assistance; through providing patients with a copy of the evidence based self-help approach reliance on health professionals may be reduced, saving time and costs for both the consumer and the health professional. In addition, individuals would have the flexibility to access the intervention program at a time of their own convenience, and to view the presentation using multiple devices such as a computer and laptop.

### Endnote

^a^Requests for the complete intervention content should be addressed to Anne Hattar, Health Psychology and Behavioural Medicine Research Group, School of Psychology and Speech Pathology, Faculty of Health Sciences, Curtin University, Western Australia (anne.hattar@curtin.edu.au).

## Appendix A: complete list of inclusion and exclusion criteria

### Inclusion criteria

Participants must be between the age of 18 to 65 years old, and overweight or obese (Body Mass Index between: 25 to 40). The individual must not have been involved in any other weight loss studies in the last 6 months, and will need the ability to restrict dietary intake, and increase physical activity levels. A reliable mobile phone with text messaging ability features is needed. The participant is required to attend the Clinic in Perth and attend all data collection time periods. Participants will be expected to complete the questionnaires and a record of physical activity and food intake for a 3 day periods when required. Participants will need to consent to blood samples, and the DEXA body composition scan.

### Exclusion criteria

Females who are pregnant, trying to become pregnant, or lactating will be excluded from the study. Participants who are diagnosed or believe they may have severe depression, eating disorders (bulimia or binge eating), health issues, and/or major illnesses/diseases will also be excluded. Major illnesses/diseases in the context of the present study includes any disease, illness, or chronic condition that may inhibit or negatively impact on participants’ ability to safety engage in a minimal contact/support intervention, or engage in a low calorie diet and physical activity intervention. Example conditions are: diabetes, heart disease, cancer, or some mental health conditions that would inhibit the participant’s health or ability to engage in the program safely with minimal support and supervision. Participants on anti-depressant medication may be excluded. In addition, any individual with past major operations in the last 2 years, or with any upcoming major operations, or cardiovascular events in the last 6 months will be excluded. Participants who consume opioids or regularly have more than two alcoholic drinks per day will also be excluded.

## Abbreviations

ANOVAS: Analysis of covariance
ANZCTR: Australian New Zealand Clinical Trial Registry
BMI: Body mass index
BMR: basal metabolic rate
DASS-21: Depression anxiety stress scales-21
DBP: Diastolic blood pressure
DEXA: Dual-energy X-ray absorptiometry
HAPA: Health action process approach
HEALTHI: Healthy eating and active lifestyle health intervention
HDL: High-density lipoprotein
IPAQ: International physical activity questionnaire
IWQOL-Lite: The impact of weight on quality of life questionnaire
LDL: Low-density lipoprotein
MANCOVA: Mixed model multivariate analyses of covariance
SBP: Systolic blood pressure
TFEQ-R18: Three factor eating questionnaire
TG: Triglycerides
